# Levels of CMV Specific CD4 T Cells Are Dynamic and Correlate with CMV Viremia after Allogeneic Stem Cell Transplantation

**DOI:** 10.1371/journal.pone.0003634

**Published:** 2008-11-04

**Authors:** Thomas Widmann, Urban Sester, Barbara C. Gärtner, Jörg Schubert, Michael Pfreundschuh, Hans Köhler, Martina Sester

**Affiliations:** 1 Department of Internal Medicine I, University clinics of the Saarland, Homburg, Germany; 2 Department of Internal Medicine IV, University clinics of the Saarland, Homburg, Germany; 3 Department of Virology, University clinics of the Saarland, Homburg, Germany; Cambridge University, United Kingdom

## Abstract

Cytomegalovirus (CMV) infection is the most frequent viral complication in patients after allogeneic stem cell transplantation. As CMV replication is tightly controlled by the cellular arm of specific immunity, the kinetics of CMV-specific T cells in association with individual reactivation episodes were prospectively analyzed in 40 allogeneic transplant recipients in a routine clinical setting and evaluated as determinant of impaired CMV control. Antigen-specific CD4 and CD8 T cells were quantified directly from whole blood using intracellular cytokine staining after specific stimulation and MHC class I multimers, respectively. Highly dynamic intraindividual changes of CMV-specific CD4 T cells were observed in patients experiencing CMV viremia. Episodes of CMV reactivation were associated with a drop of CMV-specific CD4 T cells that re-increased after viral clearance (p<0.0001). Furthermore, levels of CMV-specific CD4 T cells at the onset of viremia inversely correlated with peak viral load thereafter (p = 0.02). In contrast, CMV-peptide specific CD8 T cells did not show any association with viremia (p = 0.82). Interestingly, therapeutic dosages of cyclosporine A and corticosteroids led to a dose-dependent reduction of CMV-specific T-cell functions, indicating a causal link between intensified immunosuppressive treatment and CMV reactivation. In conclusion, levels of CMV-specific CD4 T cells inversely correlate with reactivation episodes and may represent a valuable measure to individually guide antiviral therapy after stem cell transplantation.

## Introduction

CMV specific T-cell immunity represents the key guardian for the prevention of CMV reactivation. Impaired immune reconstitution of CMV specific T cells after allogeneic stem cell transplantation disturbs this otherwise effective equilibrium between host defense and viral replication thereby contributing to an increased susceptibility to CMV replication and potentially life threatening CMV disease [1]–[5]. Although the elimination of CMV infected cells is directly mediated by functionally active CMV specific CD8 T cells [6]–[8], the need for CMV specific CD4 T-cell help to maintain or even promote the generation of virus-specific CD8 T cells has clearly been demonstrated in observational studies [9] and interventional trials of adoptive T-cell transfer in stem cell transplant recipients [10]–[13].

In recent years, intracellular cytokine induction and MHC class I multimer technology for the detection of CMV specific CD4 and CD8 T cells [1], [3], [7], [8], [10], [14]–[16], respectively, have largely replaced traditional cytotoxicity and lymphoproliferation assays [6], [9], [11], [17] with the advantage of a rapid and sensitive quantitation and functional characterization of antigen-specific T cells. These technical advances have now opened novel opportunities to assess the individual immunocompetence towards CMV in a routine clinical setting [18]. We have previously shown, that a whole CMV antigen-based stimulation of CMV specific CD4 T cells allowed for a very sensitive and specific identification of the individual CMV status [19]. Moreover, a progressive functional impairment [20] and a decrease in the level of CMV specific CD4 T cells correlated with impaired CMV control in patients after renal, heart and lung transplantation [21], [22]. Thus, quantitation of CMV specific T cells allowed the identification of patients with sufficient, insufficient or absent T-cell immunity and may therefore serve as diagnostic tool to facilitate decisions on antiviral therapy. Several promising studies suggest that similar immunological control mechanisms may play a role in patients after hematopoietic stem cell transplantation [8], [9], [15], [23], [24]. However, currently available studies were either restricted to single antigenic epitopes or a subgroup of patients with a certain HLA type. Moreover, in most studies, CMV specific T-cell immunity was analyzed far less frequently as compared to routine viral load monitoring that is usually performed on a weekly or biweekly basis.

In this study the relationship between CMV specific CD4 and CD8 T-cell immunity and CMV replication was prospectively investigated in a routine clinical setting in patients after allogeneic stem cell transplantation. Similar to our findings in renal transplant recipients, CMV specific CD4 T-cell levels showed considerable fluctuations in viremic patients and inversely correlated with CMV load. In contrast, respective T-cell frequencies in non-viremic individuals were rather stable. Together with the fact that CMV specific CD8 T cells enumerated using MHC class I pentamers did not show any association with viral load, these findings emphasize the potential use of CMV specific CD4 T-cell monitoring to identify patients at risk for viral complications and to facilitate decisions on antiviral therapy.

## Results

### Incidence of CMV viremia and acute GvHD

We have studied 40 recipients of allogeneic stem cell transplants (table 1) of whom 35 patients (87.5%) reconstituted CMV specific T-cell immunity after transplantation. 29 episodes of CMV viremia were observed in a total of in 21 patients (1 to 4 per patient) occurring after a median of 53 days (32–182 days) post transplantation, including two patients with CMV disease (one patient with CMV pneumonia and one patient with CMV gastrointestinal disease). Acute GvHD developed in 29/40 recipients (72.5%) after a median of 17.5 days post transplantation. Among those, 58.6% (17/29) consecutively became CMV-DNA positive. Moreover, 4/11 recipients (36.3%) without acute GvHD became viremic (p = 0.29), suggesting that factors other than acute GvHD may be relevant to promote CMV replication. In line with previous observations [16], CMV viremia developed more often in recipients of grafts from a CMV seronegative donor (9/11, 81.8%) as compared to patients receiving grafts from a CMV seropositive donor (12/29, 41.4%; p = 0.03).

**Table 1:** Patient characteristics.

Patients characteristics	total (n = 40)
Age (years; mean±SD)	44.9±13.9
Sex (male/female)	27/13
Recipient/donor CMV serostatus before transplantation	n (%)
R+/D+	22 (55)
R−/D+	6 (15)
R+/D−	12 (30)
Underlying diagnosis	n (%)
ALL	5 (12.5)
AML	17 (42.5)
CML	3 (7.5)
MDS	1 (2.5)
MM	5 (12.5)
NHL	6 (15)
osteomyelofibrosis	2 (5)
aplastic anemia	1 (2.5)
Stem cell source	n (%)
PBSC	33 (82.5)
BM	7 (17.5)
Donor type	n (%)
matched related donor	14 (35)
matched unrelated donor	26 (65)
Conditioning therapy	n (%)
reduced (Flu/BU, Flu/CY, Flu/CY/TBI)	10 (25)
myeloablative (BU/CY or CY/TBI)	30 (75)
GvHD incidence	n (%)
acute (any)	28 (70)
chronic	15 (37.5)

Abbreviations: BM, bone marrow; BU, busulfan; CY, cytoxin; FLU, fludarabin; MM, multiple myeloma; NHL, non-Hodgkin's lymphoma; PBSC, peripheral blood stem cells; TBI, total body irradiation.

### Dynamic changes in CMV specific CD4 T-cell numbers in viremic patients

**Figure 1:**
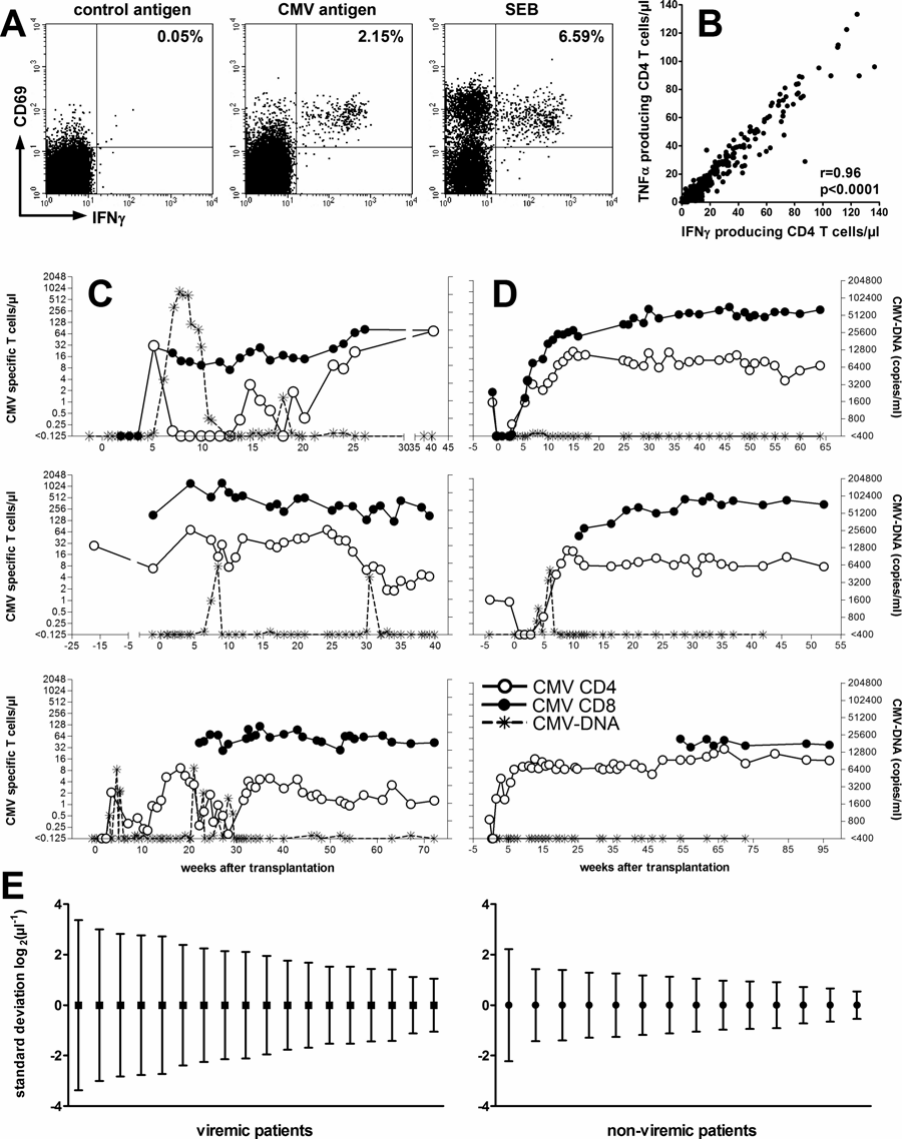
Dynamic changes of CMV specific CD4 T-cell frequencies in viremic patients. A Dotplots showing CMV specific CD4 T cells of a stem-cell transplant recipient, that were quantified by the induction of CD69 and IFN-γ after specific stimulation with CMV antigen (lysate derived from infected fibroblasts). Lysates from non-infected fibroblasts (control antigen) or *Staphylococcus aureus* enterotoxin B (SEB) served as negative and positive controls, respectively. B There was a significant correlation between the numbers of CMV specific CD4 T cells producing IFN-γ and TNF-α. Representative examples of CMV specific CD4 T cells (white circles), viral load (stars), and CMV specific CD8 T cells (black circles) over time in patients being C viremic and D non-viremic after initial CMV specific T-cell immunoreconstitution. The number of CMV specific CD8 T cells was calculated by adding T-cell levels towards individual MHC class I pentamers (between 1 and 4 per patient). E Higher standard deviations of normalized mean CMV specific CD4 T cell numbers over time in viremic patients (left panel) as compared to non-viremic patients (right panel, p<0.0001). Normalization was performed using the logarithmic values of CMV specific CD4 T cells/µl. Each symbol represents the variation in CMV specific CD4 T cells of one patient over time. Shown are all individuals where at least four samples were available over time (4-41 in viremic and 4-49 in non-viremic patients, respectively).

CMV specific CD4 T cells of stem cell transplant recipients were prospectively analyzed and correlated to CMV-DNA. CMV specific CD4 T cells were identified by upregulation of CD69 and induction of IFN-γ after stimulation with a whole CMV lysate. Stimulation with control antigen or SEB served as negative or positive controls, respectively (figure 1A). A strong correlation was found between the induction of IFN-γ and TNF-α after stimulation with CMV antigen (figure 1B, r = 0.96, p<0.0001) and SEB (r = 0.91, p<0.0001, data not shown), thus indicating a simultaneous induction of Th1 cytokines after specific stimulation.

To dissect the impact of CMV specific CD4 T cells on viral control, the number of CMV specific CD4 T cells was analyzed in viremic patients and compared to patients that did not experience viremia after CMV specific T-cell immunoreconstitution. As shown in representative examples, CMV specific CD4 T-cell levels showed a considerable fluctuation in relation to viremia (white circles in figure 1C), whereas respective T-cell levels in patients in periods of sufficient CMV control were rather stable (figure 1D). Consequently, when analyzing the variation of CMV specific T-cell levels over time, the standard deviation (SD) of normalized mean CMV specific CD4 T cells in patients with prolonged viremia varied to a larger extent (viremic, mean SD = 2.0 log_2_(µl^−1^)) as compared to recipients with stable CMV control (non-viremic, mean SD = 1.1 log_2_(µl^−1^); p<0.0001, figure 1E). Interestingly, CMV specific CD8 T-cell frequencies quantified using a panel of peptide-bound MHC class I pentamers were rather stable and did not show any fluctuations in relation to viremic episodes (black circles in figure 1B and C). As a consequence, the standard deviations of normalized mean numbers of CMV specific CD8 T cells over time did not differ between viremic and non-viremic patients (mean SD = 1.5 log_2_(µl^−1^) in viremic versus mean SD = 1.1 log_2_(µl^−1^) in non-viremic patients, p = 0.45, data not shown). Thus, dynamic changes in absolute levels of CMV specific CD4 T cells are characteristic features of patients experiencing CMV replication.

**Figure 2:**
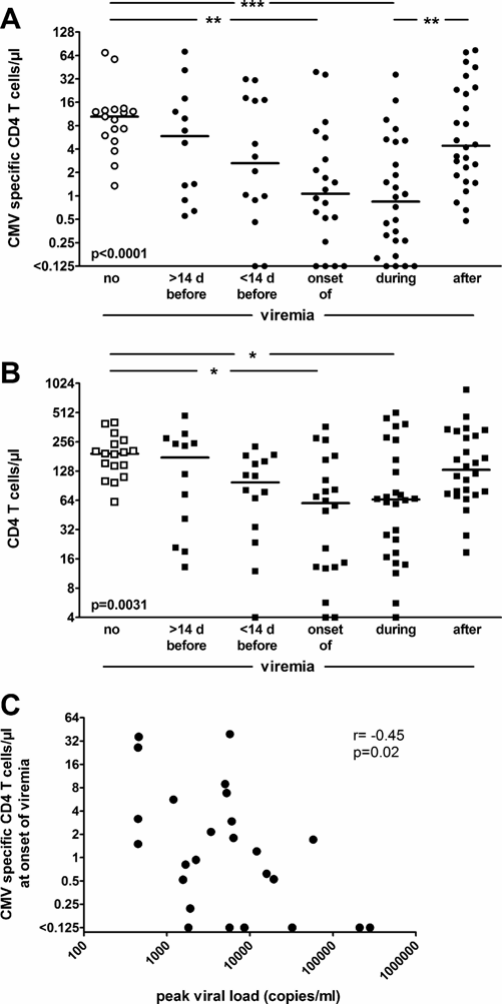
Lowest levels of CMV specific CD4 T cells at the onset of and during viremia. A CMV specific or B total CD4 T cells were quantified in non-viremic individuals and compared to patients >14 days, <14 days, at the onset of, during, or after viremia. Bars indicate median numbers of cells/µl whole blood. Each patient is depicted once in a given time period. If more than one data set existed, mean values were calculated for each patient. The level of significance in the post-test (p<0.05, p<0.01, p<0.001) is depicted by one, two or three stars, respectively. C Inverse correlation between the levels of CMV specific CD4 T cells at onset of viremia and peak viral load thereafter (r = −0.45, p = 0.02).

To more closely address the changes in CMV specific T-cell immunity in relation to viral load over time, CMV specific CD4 T-cell numbers were analyzed in patients at various stages before, during and after viremia. The group of transplant recipients who were never viremic served as a control population (figure 2A, white circles). Median CMV specific CD4 T-cell numbers in non-viremic patients reached 10.49 cells/µl (range 1.35–69.73 cells/µl). In contrast, CMV specific CD4 T-cell levels at the onset and during viremia were significantly lower (1.07 cells/µl, range 0.13–39.31 cells/µl and 0.85 cells/µl, range 0.13–36.33 cells/µl, respectively) and re-increased after resolution of viremia (4.40 cells/µl, range 0.48–74.81 cells/µl). Interestingly, CMV specific T-cell levels already started decreasing before detection of viral load (>14 days before viremia: 5.87 cells/µl, range 0.55–71.91 cells/µl; <14 days before viremia: 2.64 cells/µl; range 0.13–31.78 cells/µl). This indicates that CMV specific CD4 T-cell levels closely correlate with viral replication and may even serve as a measure to predict viremia well before its onset. Interestingly, similar kinetics were observed when the time course of viral load was related to the median number of total CD4 T cells irrespective of specificity (figure 2B). While these findings may indicate some diagnostic value of total CD4 T-cell levels as a rough surrogate parameter to judge the individual immunocompetence towards CMV, the decrease upon viremia, however, was far less pronounced as compared to that observed for CD4 T cells specific for CMV (3.0fold decrease in median CD4 T cells vs. 12.4fold decrease in median CMV specific CD4 T cells between non-viremic patients and patients during viremia).

To analyze a potential association between the level of CMV specific T cells and its impact on viral control, the number of CMV specific CD4 T cells at the onset of viremia (±3 days) was related to the peak CMV copy number during viremia in a total of 25 infectious episodes (in 4 episodes, no CMV specific CD4 T-cell analysis was performed within the ±3 days period of the onset of viremia). Interestingly, high numbers of CMV specific CD4 T cells inversely correlated with peak CMV-DNA copy number during viremia (r = −0.45, p = 0.02, figure 2C). Thus, although correlation was only moderately tight, this indicates that the counts of CMV specific T cells at the onset of viremia critically impinge on the extent of CMV replication thereafter.

### Combined immunosuppression with cyclosporine A and methylprednisolone contributes to a decrease in CMV specific CD4 T-cell function

**Figure 3:**
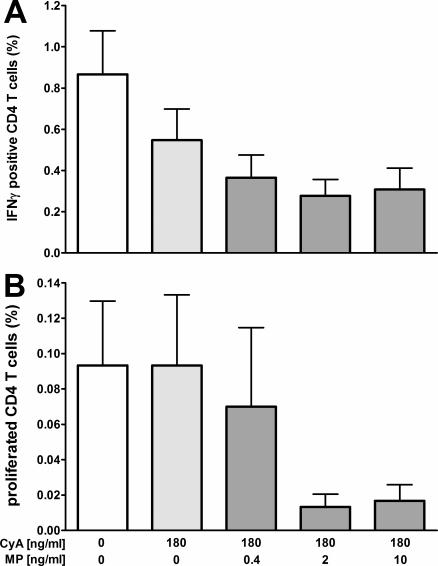
Combined immunosuppression with cyclosporine A and methylprednisolone contributes to a decrease in CMV specific CD4 T-cell function. CMV specific CD4 T cells reactivity from six immunocompetent CMV seropositive individuals were analyzed directly from whole blood supplemented with or without 180 ng/ml cyclosporine A (CyA) in the absence or presence of increasing dosages of methylprednisolone (MP). A The mean percentage of IFN-γ producing CD4 T cells (including standard error of the mean, SEM) and B the mean percentage of BrdU positive, proliferating CD4 T cells (including SEM) was analyzed after 36 h of stimulation using flow-cytometry.

Stem cell transplant recipients who experience acute GvHD receive corticosteroids in addition to their established cyclosporine A (CyA) based immunosuppressive drug regimen. As this intensified treatment may favour viral replication, the direct impact of therapeutic levels of these immunosuppressive drugs on antigen-specific effector functions was analyzed in a whole blood test (figure 3, n = 6 healthy CMV seropositive individuals). The suppressive effect of 180 ng/ml cyclosporine A in the presence or absence of 0.4, 2, or 10 ng/ml of methylprednisolone (MP, equivalent to 0.4 mg/kg, 2 mg/kg and 10 mg/kg) was analyzed on CMV specific induction of IFN-γ (figure 3A) and on CMV specific T-cell proliferation (figure 3B) after 36 h of stimulation. Incubation with cyclosporine A reduced the percentage of IFN-γ positive CD4 T cells from 0.87±0.52% to 0.55±0.37%. The addition of methylprednisolone led to a further decrease in a dose dependent manner (0.37±0.27%, 0.28±0.20%, and 0.31±0.25% for 0.4, 2 and 10 ng/ml MP; p = 0.007, figure 3A). Similarly, the percentage of BrdU positive T cells after CMV specific stimulation was reduced upon incubation with CyA and MP, although this reduction did not reach statistical significance (figure 3B). Together these data illustrate that immunosuppressive drugs and drug combinations in clinically relevant concentrations contribute to a direct suppression of T-cell function.

### Levels of CMV peptide specific CD8 T cells do not correlate with viremia

**Figure 4:**
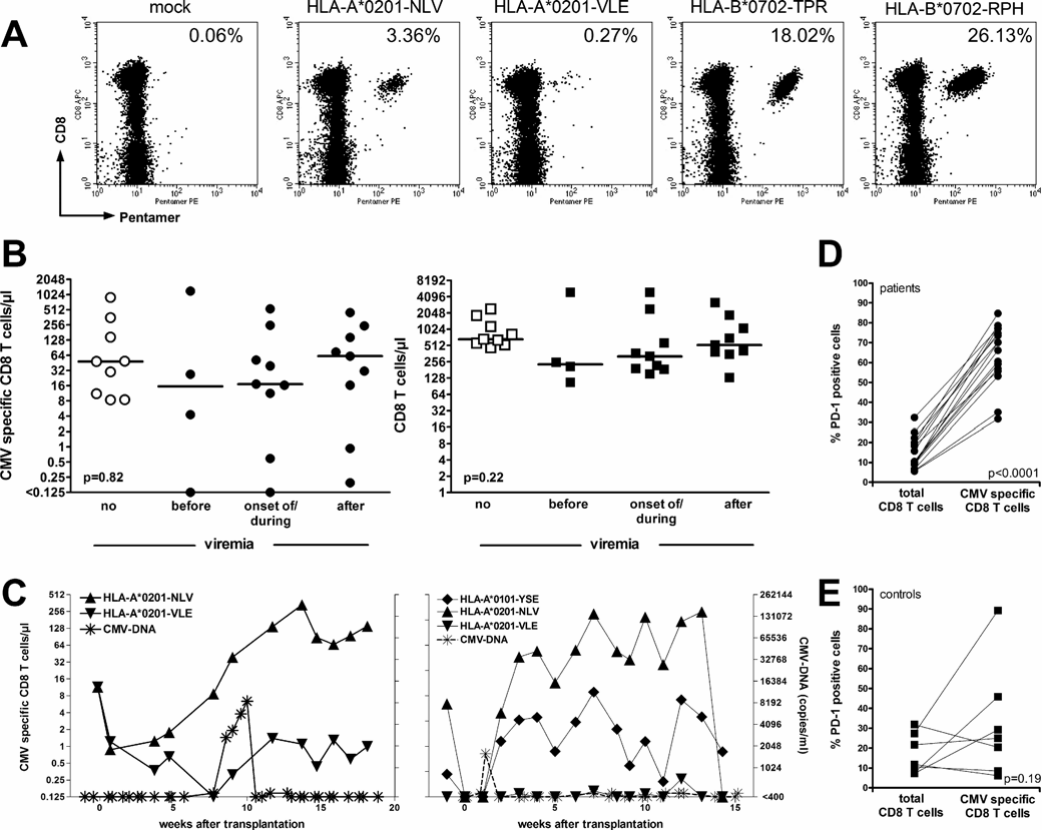
Levels of CMV peptide specific CD8 T cells do not correlate with viremia. CMV specific CD8 T cells were quantified using peptide bound MHC class I pentamers. A Dotplots of an HLA-A2 and HLA-B7 positive patient with T cells reactive towards a total of four peptides are shown. B No association of CMV specific CD8 T cells or total CD8 T cells with the presence or absence of detectable viremia (p = 0.82 and p = 0.22, respectively). Bars indicate median T-cell numbers/µl. C CMV specific CD8 T-cell numbers towards individual peptides may show different kinetics over time within one individual. D Higher percentage of PD-1 expressing CD8 T cells in CMV specific CD8 T cells (recognizing HLA-A2 with peptide NLV and/or VLE) as compared to total CD8 T cells (p<0.0001). E No significant difference in the percentage of PD-1 expressing CD8 T cells in CMV specific CD8 T cells (recognizing HLA-A2 with peptide NLV) as compared to total CD8 T cells (p<0.19).

Having shown a significant association of a drop in CMV specific CD4 T cells with an increase in viremia as compared to a considerable stability of CMV specific CD8 T-cell levels, the respective relationship of CMV specific CD8 T cells with viral replication was analyzed in a subpopulation of 15 transplant recipients with a panel of five CMV peptide-bound MHC class I pentamers. Based on the requirement for matching of the individual HLA status, between one and four different pentamer-peptide complexes could be analyzed per patient. Typical dotplots of pentamer stainings of CD8 T cells of an HLA-A2 and HLA-B7 positive patient are depicted in figure 4A (see also patient in figure 1C, middle panel). Although fewer episodes of CMV viremia (n = 9) have been studied using MHC class I pentamers, neither CMV specific nor total CD8 T-cell numbers showed any significant decrease upon viremia (figure 4B, p = 0.82 and 0.22, respectively). Moreover, as shown for two patients recognizing two and three peptides, respectively, CMV specific CD8 T-cell responses to single pentamer-peptide complexes within one patient may show profound differences in kinetics over time (figure 4C). Thus examples exist, where the CD8 T-cell responses towards single peptides are continuously rising prior to onset of viremia and thereafter (see left panel, HLA-A*0201-NLV), whereas the response towards other peptides decreases before viremia and increases thereafter (peptides HLA-A*0201-VLE or HLA-A*0101-YSE). Together this indicates that monitoring of CMV specific CD8 T cells using MHC class I multimer staining may be of limited use to identify patients at risk for viral reactivation. Interestingly, however, although numerically stable, CMV specific CD8 T cells in SCT patients exhibit some signs of functional impairment, as the expression of the programmed death 1 (PD-1) receptor on CMV specific CD8 T cells was significantly higher as compared to the whole CD8 T-cell population (Figure 4D, mean values of PD-1 expression on CD8 T cells recognizing the two HLA-A2 binding peptides versus PD-1 expression on whole CD8 T cells, p<0.0001). In contrast, there was no significant difference between the percentage of PD-1 expressing CMV specific CD8 T cells and PD-1 positive pan T cells in healthy individuals (Figure 4E, p = 0.19).

## Discussion

CMV is among the most frequent causes of infectious complications after allogeneic stem cell transplantation [2], [4], [5]. To our knowledge, this is the first study to prospectively analyze the linked dynamics of CMV specific T cells and viral load in a routine clinical setting. The incidences of CMV viremia and acute GvHD in our cohort were comparable to other previously published studies [8], [9]. In line with previous reports [8], [15], [23], a high proportion (87.5%) of patients were found to reconstitute CMV specific CD4 T-cell immunity after transplantation. Interestingly, when analyzing CMV specific CD4 T-cell dynamics over time, viremic patients showed considerable intraindividual variations, whereas CMV specific CD4 T-cell frequencies in non-viremic patients were rather stable. These dynamic changes were closely associated with viral load in that lowest frequencies of CMV specific CD4 T cells were found at the onset of and during viremia. Moreover, frequencies of specific CD4 T cells at the start of viremia inversely correlated with peak viral load thereafter. These findings are well in line with our previous observations in solid organ transplant recipients, where a drop in CMV specific CD4 T-cell frequencies correlated with increased viral replication and stable frequencies were associated with efficient viral control [21], [22].

Several factors may contribute to the decrease in CMV specific CD4 T cells over time. As intensified immunosuppressive treatment of acute GvHD is often associated with an increased incidence of CMV viremia [1], immunosuppressive drugs may directly affect CMV specific T-cell frequencies and/or –reactivity [8]. This is supported by our observation that clinically relevant concentrations of cyclosporine A and corticosteroids that were added directly to whole blood led to a dose-dependent decrease in both cytokine production as well as proliferation of CMV specific T cells. Thus, CMV specific T cells may be functionally impaired thereby contributing to an altered balance between specific immunity and viral replication [20]. This increase in viral replication may in turn lead to a progressive decrease of specific T cells over time. This decrease may be a result of both an increased recruitment to the sites of infection as well as a drug-related decrease in the proliferative capacity of CMV specific CD4 T cells. Interestingly, levels of total CD4 T cells irrespective of specificity were also lowest in patients at the onset of and during viremia. Although this may indicate some diagnostic value of total CD4 T-cell numbers as a rough surrogate parameter for the individual immunocompetence towards CMV, the difference between non-viremic and viremic patients was far more pronounced for the subset of CD4 T cells specific for CMV.

Functionality of antigen-specific T cells seems to be an essential prerequisite for efficient viral control [25]. When comparing kinetics of CMV specific CD4 and CD8 T cells, there was no association between the level of CMV specific CD8 T cells and viral load. In this study, CMV specific CD4 T cells were functionally identified by the specific activation and induction of INF-γ or TNF-α after a 6-hour stimulation. The use of a whole antigen lysate allows for a very sensitive and specific detection of CMV specific CD4 T cells independent of the patients HLA status [19]. Recently, stimulation by the use of in vitro generated autologous dendritic cells infected with CMV has been evaluated as an elegant means to quantify CMV specific T cells from stem cell transplant recipients in clinical routine [23], [24]. While this approach is attractive as it allows for a simultaneous quantitation of CMV specific CD4 and CD8 T cells, it seems more time consuming due to the need of cell purification, generation and infection of dendritic cells, and overnight stimulation. In this study, CMV specific CD8 T cells were quantified by the use of peptide-bound MHC class I multimers. Although this approach represents the most rapid technology to enumerate antigen specific CD8 T cells [18], restriction to certain HLA types and requirements for knowledge of peptide epitopes precludes the widespread use of MHC class I multimers in a routine clinical setting. Some patients may even show intraindividually different dynamics in CD8 T cells specific for individual peptide epitopes (figure 4C). Moreover, the lack of association between CMV specific CD8 T-cell frequencies and viral load indicates its limited use as parameter to identify patients at risk for viral reactivation. This may mainly be due to the fact that MHC class I multimer staining enumerates CMV specific CD8 T cells without addressing functionality. Thus, it is tempting to speculate whether a drop in functionally active CD8 T cells would be indicative of viremia. In this respect, it has indeed been shown that most CMV specific CD8 T cells in patients with reactivation are functionally impaired [24], especially during episodes of GvHD with high-level corticosteroid therapy [8]. In line with this evidence of functional anergy, we found a significantly increased expression of the PD-1 molecule on CMV specific CD8 T cells as compared to the total fraction of CD8 T cells. This molecule has recently been shown to be expressed on anergic T cells towards various chronic viral pathogens such as HIV [25], HCV [26], or LCMV in mice [27]. Thus, as with other chronic infections, efficient control of CMV replication seems to critically depend on the extent of functional anergy of antigen-specific T cells [20]. Ultimately, severe functional anergy and/or total loss of functional T cells may eventually contribute to uncontrolled virus replication and development of disease [20], [21]. Interestingly, functional anergy of both CMV and LCMV specific, PD-1 positive T cells was shown to be reverted upon blockade of PD-1 with its ligands [20], [27]. As this was associated with improved control of viral replication in vivo [27], the manipulation of antigen-specific T cells in vitro or in vivo will represent a novel therapeutic approach to improve T-cell function in chronic viral infections.

In the era of improved patient management and effective antiviral therapy [28], the occurrence of early CMV disease complications has dramatically declined and is often delayed towards later time points [9]. These late reactivations events have a high mortality [9] and are difficult to manage in a routine ambulatory setting. Our study on the linked kinetics of CMV specific CD4 T cells and viral load suggests that the monitoring of CMV specific CD4 T cells may represent a valuable approach to identify individual reactivation events in patients after CMV specific immunoreconstitution. As CMV specific CD4 T cells already started decreasing when viral load was still below detection limit, specific T-cell monitoring may even help to identify patients at risk for viral reactivation well before onset of viremia. So far, most studies that analyzed CMV specific T-cell responses after stem cell transplantation were limited by the use of single antigenic epitopes or HLA restriction as well as by considerably broad study intervals where individual reactivation episodes may not always have been closely accompanied by concomitant T-cell analyses. The technology used in this study can be widely applied in a routine clinical setting, as it can be performed from low volumes of whole blood within one day and no restriction for HLA status or knowledge of antigenic epitopes is required. Applications are widespread and could allow for an individualized targeting and timing of antiviral therapy that may avoid overtreatment and attendant toxicities [29], [30]. Although preemptive therapy in our study was guided by CMV load only, the correlation between the level of CMV specific CD4 T cells at the onset of viremia with peak viral load thereafter suggests, that CMV specific CD4 T-cell frequencies may be applied to guide both the initiation or deferral of antiviral therapy. Indeed, as shown for renal transplant recipients, stable levels of CMV specific T cells were not only typical for non-viremic patients but may also be used to identify patients, where antiviral therapy may be deferred despite detectable viral load [21]. A similar strategy was recently applied in a small series of allogeneic stem cell transplant recipients where the decision to defer antiviral therapy was based on the presence of CMV specific immunity post transplant [31]. None of the patients developed CMV disease. In this study, however, T-cell levels were only determined at 3, 4.5 and 6 months after transplantation. Given the intraindividual dynamics of CMV specific T cells, it is tempting to speculate whether potential overtreatment could further be minimized by a therapeutic decision that is based on the determination of CMV specific CD4 T-cell immunity at the onset of viremia. Similarly, as a treatment-related decrease in viral load was associated with an increase in CMV specific CD4 T cells, the duration of antiviral drug therapy may be guided by the individual recovery of CMV specific CD4 T cells thereby limiting side effects of prolonged antiviral drug exposure.

In conclusion, CMV specific CD4 T-cell levels show an inverse association with CMV viremia. Monitoring of CMV specific CD4 T cells on a routine basis may help to predict uncontrolled CMV viremia after allogeneic stem cell transplantation and may in future be used as important clinical tool to identify both high-risk patients and patients who may not need antiviral therapy. Clearly, this should be explored in a prospective randomized clinical trial as it is currently being performed in patients after renal transplantation in our transplant center.

## Materials and Methods

### Patient characteristics

A total of 40 allogeneic transplant patients were recruited from October 2002 to September 2005. Patient characteristics and demographic data are shown in table 1. Monitoring for CMV-DNA from peripheral blood was performed weekly or biweekly during inpatient treatment and at outpatient visits thereafter. Antiviral treatment included prophylactic application of acyclovir and preemptive therapy with ganciclovir or foscarnet. Monitoring of CMV-specific T cells from 1.5 ml of whole blood was performed weekly during inpatient treatment and at outpatient visits thereafter. CMV disease was defined according to established criteria [32]. Six CMV seropositive healthy control persons (39.0±5.5 years of age) were recruited to analyze the effect of immunosuppressive drugs on T-cell reactivity and proliferation in vitro. Seven CMV seropositive HLA-A2 positive healthy control persons (39.8±14.7 years of age) were analysed for PD-1 expression on CMV specific CD8 T cells. The study was approved by the local ethics committee (“Ethikkommission der Ärztekammer des Saarlandes”) and all patients gave informed consent. Consent to use 1.5 ml of blood for the study was obtained on a verbal basis as blood was drawn upon clinically indicated venipunctures only.

### Quantitation of CMV specific CD4 T cells

Quantitation of CMV specific CD4 T cells was performed directly from whole blood (450 µl/sample) as described before [19], [21], [22]. Briefly, titered amounts of CMV antigen (32 µl/ml; Virion, Würzburg, Germany) were used as stimulus in the presence of 1 µg/ml anti-CD28 and anti-CD49d (clones L293 and 9F10; BD, Heidelberg, Germany), respectively, to induce antigen-specific activation and cytokine induction. As negative and positive controls, cells were stimulated with control antigen (Virion), and 2.5 µg/ml *Staphylococcus aureus* Enterotoxin B (SEB, Sigma, Deisenhofen, Germany), respectively. Blood was incubated in 15 ml-polypropylene tubes at 37°C at 6% CO_2_ for a total of 6 h. During the last 4 h 10 µg/ml of brefeldin A (Sigma) was added to block secretion of cytokines. Thereafter the blood was treated with 2 mM EDTA for 15 minutes. Subsequently, erythrocytes were lysed and leukocytes were fixed for 10 minutes using BD lysing solution (BD). Cells were washed once with FACS buffer (PBS, 5% filtered FCS, 0.5% BSA, 0.07% NaN_3_) and immunostained in FACS buffer containing 0.07% saponin (Sigma) using anti-CD4 (clone SK3), anti-CD69 (clone L78), anti-IFNγ (clone 4S.B3), anti-TNFα (clone MAb11, all antibodies from BD). Samples were analyzed on a FACS Calibur (BD) using Cellquest Pro software. Absolute numbers of CMV specific CD4 T cells/µl whole blood were calculated based on differential blood counts. To analyze the effect of immunosuppressive drugs on T-cell reactivity and proliferation, whole blood was preincubated for two hours with various dosages of steroids and/or cyclosporine A and stimulated with CMV antigen for a total of 36 hours. After 6 hours, an equal volume of RPMI supplemented with 1% glutamine, 1% penicillin-streptomycin, and 0.5% human serum albumine was added. Proliferation was assessed by incorporation of 500 µM bromo-desoxy uridine (BrdU, Sigma) that was added after 28 hours. The last 4 hours of incubation were carried out in the presence of 10 µg/ml brefeldin A. Thereafter, samples were treated as described above and stained for CD4, IFN-γ and BrdU (clone B44, BD).

### Quantitation of CMV specific CD8 T cells

Quantitation of peptide-specific CD8 T cells from HLA-A*0101, HLA-A*0201, and/or HLA-B*0702 positive patients was performed from whole blood using the following MHC class I pentamers (bound peptide sequences and amino acid positions within pp65 are shown in brackets): HLA-A*0101-YSE (YSEHPTFTSQY, HCMV pp65 363–373), HLA-A*0201-NLV (NLVPMVATV, HCMV pp65 495–504), HLA-A*0201-VLE (VLEETSVML, HCMV IE1 316–324), HLA-B*0702-TPR (TPRVTGGGAM, HCMV pp65 417–426), HLA-B*0702-RPH (RPHERNGFTVL, HCMV pp65 265–275). Staining was performed according to the manufacturer's instructions (ProImmune, Oxford, UK). Pentamer binding was visualized using anti-Pentamer PE and cells were co-stained using CD8 APC (clone SK1, BD). PD-1 expression on CMV specific CD8 T cells from HLA-A*0201 positive patients was assessed from leukocytes frozen over time and analyzed as a batch. PD-1 (clone MIH4, FITC, BD) was stained together with CD8 (clone SK1, APC) and HLA-A*0201-tetramers bound to peptides NLVPMVATV and VLEETSVML (Orpegen Pharma, Heidelberg, Germany).

### Determination of CMV serostatus and viral load

The CMV serostatus was determined by a commercial CMV IgG test (CMV IgG PKS, medac, Hamburg, Germany). CMV load (CMV-DNA) was measured using the Cobas Amplicor assay (Roche Diagnostics, Mannheim, Germany).

### Statistical analysis

Statistical analysis was performed using Prism V4.03 software (Graphpad, San Diego, USA). Fisher's exact test was used to associate CMV reactivation episodes to graft-versus-host disease (GvHD) or to CMV seroconstellation. The unpaired t-test was used to compare the standard deviations of individual CMV specific CD4 and CD8 T-cell frequencies over time in viremic and non-viremic individuals. The non-parametric Kruskal Wallis test with Dunn's multiple comparison test was used to compare CMV specific CD4 and CD8 T-cell levels in non-viremic patients and patients before, during and after viremia. The correlation between CMV specific CD4 T-cell levels at onset of viremia and peak CMV-DNA copy number thereafter, and the correlation between the frequencies of CMV or SEB reactive CD4 T cells expressing IFN-γ or TNF-α was calculated according to Spearman. The effect of immunosuppressive drugs on CMV specific T-cell reactivity was analyzed using the repeated measure ANOVA with Bonferronis multiple comparison test. The difference between the percentage of PD-1 expressing CMV specific CD8 T cells and pan CD8 T cells was calculated using the paired t-test.
